# Parsing the Kinetic Energy Budget of the Ocean Surface Mixed Layer

**DOI:** 10.1029/2021GL095920

**Published:** 2022-01-18

**Authors:** Seth F. Zippel, J. Thomas Farrar, Christopher J. Zappa, Albert J. Plueddemann

**Affiliations:** ^1^ Woods Hole Oceanographic Institution Falmouth MA USA; ^2^ Lamont‐Doherty Earth Observatory Columbia University Palisades NY USA

**Keywords:** air/sea interaction, turbulence, mixed layer, wind work, boundary layer, waves

## Abstract

The total rate of work done on the ocean by the wind is of considerable interest for understanding global energy balances, as the energy from the wind drives ocean currents, grows surface waves, and forces vertical mixing. A large but unknown fraction of this atmospheric energy is dissipated by turbulence in the upper ocean. The focus of this work is twofold. First, we describe a framework for evaluating the vertically integrated turbulent kinetic energy (TKE) equation using measurable quantities from a surface mooring, showing the connection to the atmospheric, mean oceanic, and wave energy. Second, we use this framework to evaluate turbulent energetics in the mixed layer using 10 months of mooring data. This evaluation is made possible by recent advances in estimating TKE dissipation rates from long‐enduring moorings. We find that surface fluxes are balanced by TKE dissipation rates in the mixed layer to within a factor of two.

## Key Points

- We estimate terms in the vertically integrated turbulent kinetic energy (TKE) equation from 10 months of mooring data, including TKE dissipation rates
- A conceptual framework is developed to separate the shallow wave‐affected layer from the mixed layer below
- Applying this framework to the TKE budget, we find that measured dissipation rates balance surface‐driven forcing to within a factor of two

## 1 Introduction

The input of mechanical energy to the ocean from wind (the rate of wind work) drives ocean currents, grows surface waves, and creates turbulence that can enhance vertical mixing. Estimates of air/sea KE (Kinetic Energy) flux have been made by numerous studies using both models and global products (Ferrari & Wunsch, 2009, 2010; von Storch et al., 2007; Wunsch, 1998; and many others). The majority of these studies focus on estimates and analysis relating to the shear‐driven surface flux, the product of the surface stress and the surface current ${\left(\bar{\tau \cdot u}\right)}_{0}$, which although is simple in form, can be difficult to estimate. Direct measurement of ocean surface current and stress is challenging, and not commonly made via global or local in situ measurements. Instead, more available estimates of atmospheric boundary layer stress and geostrophic, subsurface, or drifter‐derived currents are used, each with their own set of challenges, assumptions and caveats when applied toward estimating ${\left(\bar{\tau \cdot u}\right)}_{0}$ at the ocean surface. Further, the overbar denoting a temporal average indicates that this form of the KE flux includes both mean, turbulent, and wave‐coherent components, further complicating both estimation and analysis of the surface KE flux. The fraction of total wind work that is partitioned into currents, waves, and turbulence is poorly constrained but is important in determining the energy available for driving mean currents, waves, and vertical mixing respectively.

A summary of recent best estimates of the partitioning of global air/sea KE flux is presented in Table 2 of Wunsch (2020) and shows the total wind work on the ocean is estimated at 70 TW (Ferrari & Wunsch, 2010), with 68 TW going to surface gravity waves (Rascle et al., 2008), 1–3 TW going toward general circulation (Rimac et al., 2016), and 0.2 TW going to internal waves (Thorpe, 2005). No direct estimates have been made for the amount available for turbulent energy in the upper ocean, which is related to both the wave‐mediated and viscous‐stress‐mediated work at the interface. Wave‐mediated fluxes at the surface estimated at ∼68 TW are the largest contribution, and since only a small fraction of this surface wave energy is estimated to reach the coastlines (2.4 TW, Rascle et al., 2008), the majority is expected to stay in the ocean basins and be transferred into mean currents and turbulence. Even though these wave‐driven fluxes are large, the energy they impart to the upper ocean is predominately held to a thin near‐surface region because both wave breaking turbulence (Terray et al., 1996) and Stokes drift shear decay rapidly with depth. We will herein refer to the energetic near‐surface layer as the wave‐affected layer and distinguish it from the oceanic mixed layer below. Many LES (Large Eddy Simulation) studies suggest the importance of Stokes forcing may lie in its ability to enhance downward transport, modify turbulence anisotropy, and alter the relative direction of shear to the local stress, all of which can be important for entrainment at the mixed layer base (Grant & Belcher, 2009; Large et al., 2021; Li & Fox‐Kemper, 2020; McWilliams et al., 1997).

A large body of work over the last 30 years has focused on understanding the wind work on currents at near‐inertial frequencies (Alford, 2020; D’Asaro, 1985; Plueddemann & Farrar, 2006) because near‐inertial waves can transmit energy and momentum over long distances and are important sources of shear, shear instabilities, and mixing in the ocean interior. These estimates are sensitive to the amount of kinetic energy dissipated in the mixed layer, and models that do not explicitly include turbulent mixing result in biased estimates of inertial kinetic energy (Plueddemann & Farrar, 2006). Previous methods for estimating the turbulent energy sinks in the mixed layer have been indirect, using either 1D mixed layer models such as Price et al. (1986), or assumed balances in the Turbulent Kinetic Energy (TKE) equation (Alford, 2020).

**Figure 1:**
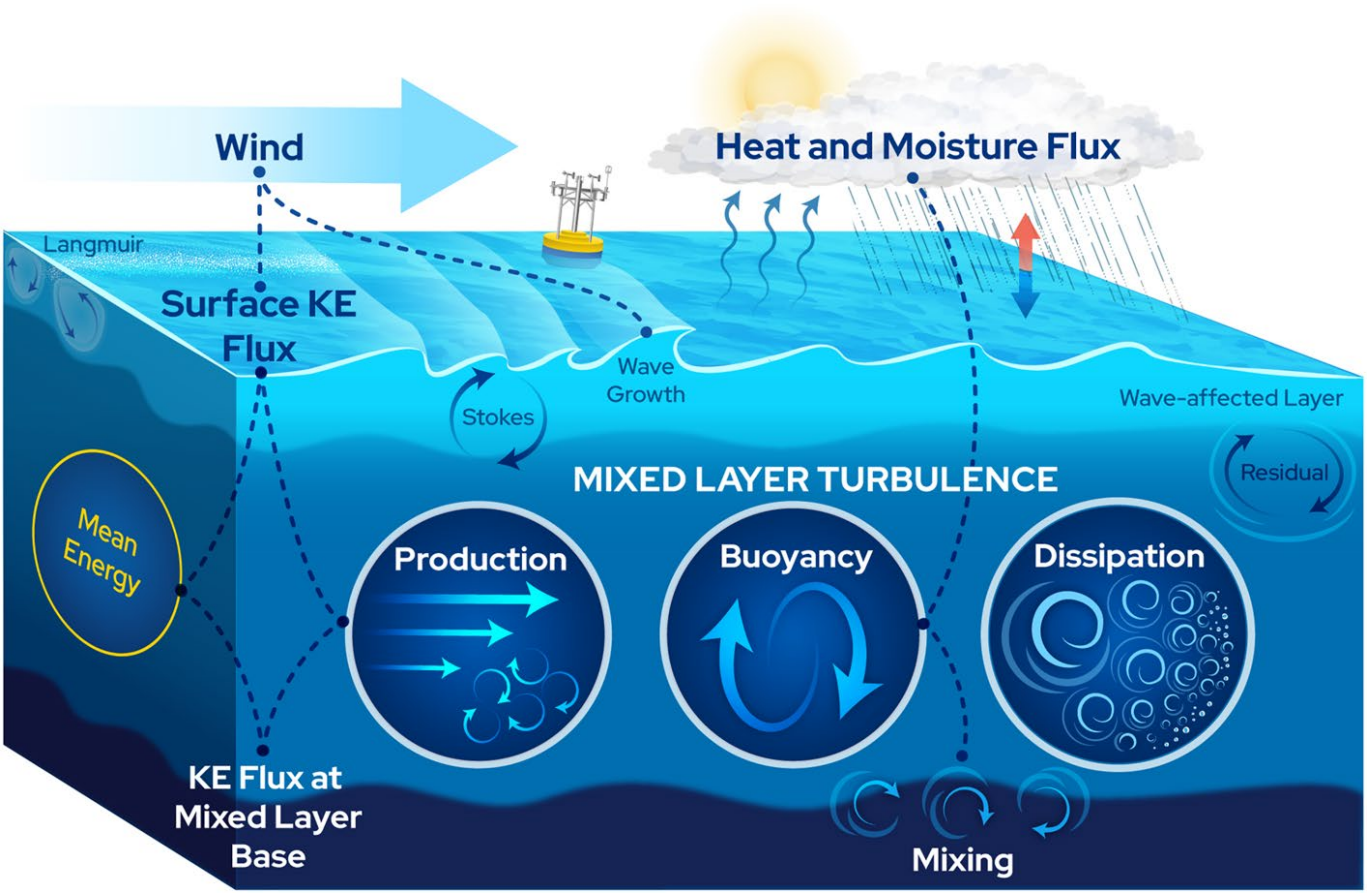
The schematic above highlights surface processes and pathways for kinetic energy (KE) transfer between the atmosphere and the ocean. Dashed lines and solid dots indicate how terms in the vertically integrated mixed‐layer TKE equation (Equation 11) connect to the atmosphere, the wave‐affected layer, the deeper ocean, and the mean KE equation. Kinetic energy fluxes from the wind are split between viscous and wave‐driven terms at the interface. The majority of wave‐supported energy fluxes balance with terms in the wave‐affected layer. Here, we focus on the balance in the mixed‐layer, where surface‐driven production and buoyancy are primarily balanced by TKE dissipation rates.

Here, we examine turbulent energy losses in the oceanic mixed layer directly using a 10‐month time series of TKE dissipation rates made from an ocean mooring (S. F. Zippel et al., 2021). These estimates from long‐enduring deep‐ocean platforms showcase a powerful new approach toward understanding mixed layer dynamics in the upper 100 m of the ocean. In Section 2, we lay out a framework for analyzing the vertically integrated TKE equation and show its relation to the oceanic mean and surface gravity wave energy equations (as schematized in Figure 1). A key aspect is the separation of the wave‐affected layer from the mixed layer below. In Section 3, we overview the mooring measurements, setup, and location. The results are presented in Section 4 and discussed in Section 5.

## 2 Posing the Vertically Integrated TKE Equation

Integrating the vertical TKE equation as posed by McWilliams et al. (1997) from arbitrary depth, *z*, to the surface gives,

(1) $$\int_{z}^{0}\left[\underset{Production}{\underset{︸}{\tau \cdot \frac{d\bar{u}}{dz}}}+\underset{StokesProduction}{\underset{︸}{\tau \cdot \frac{du_{s}}{dz}}}-\underset{Buoyancy}{\underset{︸}{g\bar{w^{\prime }\rho^{\prime }}}}-\underset{Transport\;Divergence}{\underset{︸}{\frac{d}{dz}\left(\bar{p^{\prime }w^{\prime }}+\rho_{0}\bar{e^{\prime }w^{\prime }}\right)}}-\underset{Dissipation}{\underset{︸}{\rho_{0}\epsilon }}\right]dz=0,$$

where $\tau =-\rho_{0}\bar{u^{\prime }w^{\prime }}$ is the turbulent stress, **u** is the horizontal current, ***u***_*s*_ is the Stokes drift, *ρ* is the density, *p* is the pressure, *w* is the vertical velocity, *e* is the TKE, *ϵ* is the TKE dissipation rate, bold font represents a vector quantity, overlines represent Reynolds (time) averaging, and primed values denote turbulent fluctuations such that $u=\bar{u}+u^{\prime }$, and z is defined to be positive up with the surface at *z* = 0. Terms in Equation 1 are discussed separately in subsections below, and the evaluated equation is schematized in Figure 1.

### 2.1 Buoyancy Flux

Following Li and Fox‐Kemper (2017), we decompose the buoyancy flux into a component driven by surface fluxes, *B*_0_, and a component driven by mixing at the base of the mixed layer, *B*_*H*_. Li and Fox‐Kemper (2017) assumed a simply linear structure across the mixed layer such that

(2) $$-g\bar{w^{\prime }\rho^{\prime }}\approx B_{0}\left(1+\frac{z}{H}\right)-B_{H}\frac{z}{H},$$

where *H* is the mixed‐layer depth such that at the surface where $-g\bar{w^{\prime }\rho^{\prime }}(z=0)=B_{0}$ and at the base of the mixed layer where $-g\bar{w^{\prime }\rho^{\prime }}(z=-H)=B_{H}$. We note here that this assumption is more suitable for unstable surface forcing and may result in errors under strong stable conditions. The surface flux, *B*_0_, results from a combination of net radiation (short wave *Q*_*s*_ and long wave, *Q*_*l*_), latent and sensible heat fluxes (*Q*_*H*_ and *Q*_*B*_), and precipitation and evaporation, which can be expressed,

(3) $$B_{0}=-\frac{g\alpha }{c_{p}}\left(Q_{s}+Q_{l}+Q_{H}+Q_{B}\right)+g\beta \left(E-P\right)S_{0},$$

where *α* is the thermal expansion coefficient, *c*_*p*_ is the specific heat of water, *β* is the haline contraction coefficient, E and P are the rates of evaporation and precipitation, and *S*_0_ is the surface salinity. The shortwave radiation has a depth‐dependent absorption rate, and therefore is not applied solely at the surface. This can be approximated with an exponential decay of two primary wavelengths (Price et al., 1986), such that,

(4) $$Q_{s}=Q_{s}\left(z\right)\approx Q_{s,0}\left(I_{1}e^{z/\lambda_{1}}+I_{2}e^{z/\lambda_{2}}\right),$$

with *I*_1_ = 0.62, *I*_2_ = 1 − 0.62, *λ*_1_ = 0.6 m, *λ*_2_ = 20 m, and *z* ≤ 0. The vertical integral of the buoyancy flux is then,

(5) $$\int_{z}^{0}-g\bar{w^{\prime }\rho^{\prime }}dz\approx -B_{0}\left(z+\frac{z^{2}}{2H}\right)-I_{S}\left(z\right)+\frac{z^{2}}{2H}B_{H},$$

where *I*_*S*_(*z*) is the difference between the total shortwave heat flux and the integral of the shortwave decay function over coordinate *z*.

### 2.2 TKE Production

The Eulerian shear production term in Equation 1 is linked to both the production/destruction of mean energy, and the total energy. This connection can be shown through a number of mathematical identities, and here, we show the connection through applying integration by parts to the turbulent production term,

(6) $$\underset{Turbulent\;Production}{\underset{︸}{\int_{z}^{0}\tau \cdot \frac{d\bar{u}}{dz}dz}}=\underset{Surface\;KE\;Flux}{\underset{︸}{{\left(\bar{\tau \cdot u}\right)}_{0}}}-\underset{KE\;Flux\;at\;z}{\underset{︸}{{\left(\bar{\tau \cdot u}\right)}_{z}}}-\underset{Mean\;Energy\;Production}{\underset{︸}{\int_{z}^{0}\bar{u}\cdot \frac{d\tau }{dz}dz}},$$

where the subscript (⋅)_0_ represents evaluation at the surface and subscript (⋅)_*z*_ represents evaluation at depth *z*. Here, the first two terms on the RHS are the KE flux through the surface and across depth *z*, respectively. These two terms can be thought of as the total energy flux into the layer and are consistent with the similar volume‐integrated surface flux defined by von Storch et al. (2007) (Equation 10 therein). The third term on the RHS is the energy input to the depth‐integrated mean energy equation. This term also appears in the mean energy equation, which is formed by taking the dot product of $\bar{u}$ with the mean momentum equation and then vertically integrating. Equation 6 can be seen as relating the total shear‐driven KE flux in and out of the layer to the respective mean and turbulent components, and is schematically represented by the four linked components on the left side of the schematic shown in Figure 1.

Recognizing that the turbulent stress generally decreases with depth, linear decay is a reasonable first approximation for the depth dependence. We acknowledge that this linear model implies no vertical shear within the mixed layer, and therefore is not strictly correct. However, here the model is used only to decay the surface stress, and currents will be estimated with measurements (to be described in Sections 3 and 4). Therefore, we assume the difference between linearly decaying stress and a stress profile with more curvature is small in the context of this study. Applying this model, we impose a shape for the turbulent stress as *τ*(*z*) = *τ*_0_(1 + *z*/*H*) such that at the surface *τ*(0) = *τ*_0_, and at the base of the mixed layer *τ*(*z* = −*H*) = 0. The vertically integrated TKE production can be represented as,

(7) $$\int_{z}^{0}\tau \cdot \frac{d\bar{u}}{dz}dz\approx \bar{\tau_{0}\cdot u_{0}}-\left(\bar{\tau_{0}\cdot u_{z}}\right)\left(1+z/H\right)-\int_{z}^{0}\bar{u}\cdot \frac{\tau_{0}}{H}dz,$$

where ***τ***_0_ is the surface stress. The advantage of this form of the turbulent production is that it can be estimated from the surface stress, the velocity profile, and the mixed layer depth, whereas direct measurement of *τ*(*z*) and *dU*/*dz* in the upper ocean over long durations remains a significant observational challenge. Similarly, no scaling arguments have been made, although we note that for classic neutral boundary layer flows the kinetic energy flux is expected to scale as $u_{*}^{3}$, where $u_{*}=\sqrt{\tau_{0}/\rho }$.

Combining Equations 5 and 7 into Equation 1 and evaluating at *z* = −*H* gives,

(8) $$\Pi_{0}-\int_{-H}^{0}\bar{u}\cdot \frac{\tau_{0}}{H}dz+\left(\frac{B_{0}H}{2}-I_{S}\left(-H\right)+\frac{B_{H}H}{2}\right)+\int_{-H}^{0}\tau \cdot \frac{du_{s}}{dz}dz-\int_{-H}^{0}\rho_{0}\epsilon dz=0,$$

where we have assumed the pressure and TKE flux are zero at *z* = −*H*, and we have defined a surface energy flux as $\Pi_{0}=\bar{\tau_{0}\cdot u_{0}}+{\left(\bar{p^{\prime }w^{\prime }}\right)}_{0}$, which combines the surface shear and pressure fluxes (note, the TKE flux $\bar{e^{\prime }w^{\prime }}$ is assumed to be zero at the surface, and the pressure flux relating to surface wave effects will be discussed in the following section). Some definitions of Π_0_ may also include the Stokes‐driven KE flux at the surface, which we have opted to keep as a separate term here such that Π_0_ is the Eulerian KE flux. The shear‐driven flux, $\bar{\tau_{0}\cdot u_{0}}$ contains both mean, and wave‐coherent components. These wave‐coherent components of the surface fluxes are active areas of research, and so we apply our current conceptual framework to include wave effects, as schematized in Figure 1, while recognizing future research may change our understanding of how wave‐driven kinetic energy fluxes manifest in the TKE equation in the atmospheric and oceanic wave‐affected boundary layers.

### 2.3 Surface Wave Fluxes and the Wave‐Affected Layer

Waves gain energy from the atmosphere through a combination of wave‐coherent surface motions and pressures (Belcher & Hunt, 1993; Jeffreys, 1924; Miles, 1957), which continue to be an active research topic. For example, laboratory studies (M. P. Buckley & Veron, 2016; M. Buckley et al., 2020) and process studies (Grare et al., 2018) have shown the existence of wave‐coherent viscous and turbulent stresses in the wave‐affected atmospheric boundary layer. Field measurements (Donelan et al., 2005; Snyder et al., 1981) have confirmed that wave‐coherent pressure in atmosphere results in a non‐zero pressure‐mediated surface flux ${\left(\bar{p^{\prime }w^{\prime }}\right)}_{0}$ (sometimes called the pressure work, or the piston pressure). The challenge, therefore, is that the total surface KE flux at the interface, $\Pi_{0}={\left(\bar{\tau \cdot u}\right)}_{0}+{\left(\bar{p^{\prime }w^{\prime }}\right)}_{0}$, cannot be estimated as the measured wind stress and a mean surface current, $\tau_{air}\cdot {\bar{u}}_{0}$, which would misrepresent wave‐mediated transfers. Direct measurement of these fluxes at the surface is beyond the ability of standard in situ and remotely sensed products. Therefore, we seek a way to include wave‐layer effects in Equation 8 in way that can be estimated from deep‐ocean mooring measurements of wind stress, wave spectra, and currents.

Following (Gemmrich et al., 1994), we decompose the surface KE flux into viscous and wave‐coherent components such that $\Pi_{0}^{air}\approx \tau_{\nu }\cdot {\bar{u}}_{0}+F_{in}$, where *F*_*in*_ is the energy gained by the surface waves from the wind and ***τ***_*ν*_ is the viscous stress. Here, *F*_*in*_ includes the wave‐coherent components of ${\left(\bar{\tau \cdot u}\right)}_{0}$ as well as the pressure‐work, and can be parameterized using the surface wave spectrum, *E*(*ω*, *θ*) and a growth rate *β*_*w*_ as $F_{\text{in}}=\rho_{0}g\;\int \;\int \;\beta_{w}\omega E(\omega ,\theta )d\omega dd\theta$ (e.g., The WAMDI Group, 1988). Similarly, the ocean surface flux can be parameterized using a wave energy loss such that $\Pi_{0}=\tau_{\nu }\cdot {\bar{u}}_{0}+F_{ds}$, where *F*_*ds*_ is the energy loss from the surface wave field. In this way, energy is conserved at the air/sea boundary between wave and non‐wave components as $\Pi_{0}^{air}-\Pi_{0}=\Pi_{wave}=F_{in}-F_{ds}$.

Assuming an atmospheric constant stress layer, the surface viscous stress, ***τ***_*ν*_, can be related to a turbulent stress measured above the wave‐affected atmospheric layer, ***τ***_*air*_, and the wave stress, ***τ***_*wave*_, such that, ***τ***_*ν*_ = ***τ***_*air*_ − ***τ***_*wave*_. The wave stress can again be estimated using wave parameterizations, $\tau_{wave}=\rho_{0}g\int \beta_{w}kE\left(\omega ,\theta \right)\left[\cos\left(\theta \right)\hat{i}+\sin\left(\theta \right)\hat{j}\right]d\omega d\theta$, where here *k* is the wavenumber and $\hat{i},\hat{j}$ represent horizontal vector components.

Therefore, the ocean surface KE flux can be estimated as,

(9) $$\Pi_{0}\approx \tau_{\nu }\cdot {\bar{u}}_{0}+F_{ds}=\left(\tau_{air}-\tau_{wave}\right)\cdot {\bar{u}}_{0}+F_{ds}.$$

Here, it is more clear why $\tau_{air}\cdot {\bar{u}}_{0}$ is not an appropriate surface KE flux. In fact, the difference between the commonly applied KE flux and that presented here is shown to be, $\Pi_{0}-\tau_{air}\cdot {\bar{u}}_{0}=F_{ds}-\tau_{wave}\cdot {\bar{u}}_{0}$. For typical open ocean conditions, the quantity $F_{ds}-\tau_{wave}\cdot {\bar{u}}_{0}$ can be large since the wave‐energy transfer velocity scales with the phase speed, which is typically much larger than the mean surface current ${\bar{u}}_{0}$ (Gemmrich et al. (1994) defines a wave energy transfer speed as ${\bar{c}}_{p}=F_{in}/\tau_{wave}$. Using this definition, Equation 9 can be rearranged to show that for wind/wave equilibrium conditions, $\Pi_{0}-\tau_{air}\cdot {\bar{u}}_{0}=F_{ds}\left(1-{\bar{u}}_{0}/{\bar{c}}_{p}\right)$. Typical open ocean conditions would suggest ${\bar{u}}_{0}/{\bar{c}}_{p}$ of order 10^−2^ to 10^−1^, such that $\tau_{air}\cdot {\bar{u}}_{0}$ is smaller than Π_0_ by roughly *F*_*ds*_, which can be significant. We further note that ${\bar{c}}_{p}$ differs slightly from *c*_*eff*_ defined by Terray et al. (1996) as *c*_*eff*_ = *F*_*in*_/*τ*_*air*_).

It is not clear which terms in Equation 8 directly link to the wave‐driven ocean surface energy flux *F*_*ds*_. Conceptually, wave dissipation in spectral models, *F*_*ds*_, has largely been tuned to give the appropriate wave heights in models (Cavaleri et al., 2019) and can be associated with a variety of wave processes including whitecapping, microbreaking, wave‐turbulence interactions, and other hypothesized interactions. Although the mechanisms are unclear, numerous field studies have successfully linked measurements of enhanced upper ocean TKE dissipation rates to estimates of the wave‐energy loss term (Gerbi et al., 2009; Sutherland & Melville, 2015; Terray et al., 1996; Thomson et al., 2016). Models for near‐surface turbulence can produce similar decay slopes and turbulence levels with an assumed balance between the transport divergence and the TKE dissipation rate in the wave‐affected layer (Burchard, 2001; Craig & Banner, 1994). There is also some observational support that enhanced near‐surface TKE dissipation rates are balanced specifically by pressure vertical‐velocity correlations (Scully et al., 2016). Following from these observational and modeling studies, we assume that the dominant turbulent balance for the wave‐affected layer can be posed,

(10) $$\int_{z_{t}}^{0}\frac{d}{dz}\left(\bar{p^{\prime }w^{\prime }}+\rho_{0}\bar{e^{\prime }w^{\prime }}\right)dz\approx {\left(\bar{p^{\prime }w^{\prime }}\right)}_{0}=\rho_{0}\int_{z_{t}}^{0}\epsilon dz=C_{ds}F_{ds},$$

where *C*_*ds*_ is the fraction of wave dissipation that is converted to turbulence, and *z*_*t*_ is the depth of the wave‐affected layer. Measurements have shown that TKE dissipation rates are enhanced compared to rigid‐wall boundary layer scalings at depths roughly *z*_*t*_ = −10*H*_*s*,*ww*_ and above, where *H*_*s*,*ww*_ is the height of the wind waves (Gerbi et al., 2009; Terray et al., 1996). Therefore, it is reasonable to assume that below this layer, the transport divergence terms are relatively small. The fraction of wave breaking energy available for turbulence, *C*_*ds*_, is not well constrained with measurements reporting *C*_*ds*_ from 10% to 100% over a variety of wave ages and wave breaking types (Feddersen, 2012; Scully et al., 2016; Sutherland & Melville, 2015; S. Zippel & Thomson, 2015).

### 2.4 The Wave Affected Layer to the Mixed‐Layer Base

Here, we wish to investigate energetic fluxes from below the wave‐affected layer at *z*_*t*_ to the base of the mixed layer, *H*. We assume the wave‐affected balance shown in Equation 10 holds to within some small residual, and using the surface flux defined in Equation 9, with Equation 8, we integrate from the base of the wave‐affected layer, *z*_*t*_ (rather than the surface, *z* = 0), to the mixed layer depth, *H*.

(11) $$\begin{array}{l} \underset{Surface\;Production}{\underset{︸}{(\tau_{air}-\tau_{wave})\left(1+\frac{z_{t}}{H}\right)\cdot {\bar{u}}_{z_{t}}-\int_{-H}^{z_{t}}\bar{u}\cdot \frac{\tau_{air}\;-\;\tau_{wave}}{H}dz}}+\underset{Stokes\;Production}{\underset{︸}{\int_{-H}^{z_{t}}\tau \cdot \frac{du_{s}}{dz}dz}}+\\ \underset{Surface\;Buoyancy}{\underset{︸}{B_{0}\left[\frac{H}{2}+z_{t}+\frac{z_{t}^{2}}{2H}\right]-I_{S}\left(-H\right)+I_{S}\left(z_{t}\right)}}-\underset{Dissipation}{\underset{︸}{\rho_{0}\int_{-H}^{z_{t}}\epsilon dz}}=\underset{Residual}{\underset{︸}{R}}. \end{array}$$

Here, the residual term on the RHS includes the buoyancy associated with vertical mixing, *B*_*H*_, residual wave‐breaking fluxes (1 − *C*_*ds*_)*F*_*ds*_, and the pressure and KE fluxes (i.e., transport divergence terms) at *z* = *z*_*t*_ and at *z* = −*H*.

To summarize the many steps made to arrive at Equation 11, we have assumed: stationary conditions, linear decay of the ocean surface stress *τ*_0_ across the mixed layer, an exponential decay for two primary wavelengths of shortwave radiation and a linear decay for the other surface buoyancy terms, zero turbulent transport at the mixed layer base, zero TKE flux at the surface, a parameterization for wave‐driven momentum flux based on the sea surface elevation spectrum, a constant stress layer in the atmospheric boundary layer, and a transport divergence—TKE dissipation rate balance in the wave‐affected layer that closes to a small residual R above the wave‐affected layer depth, *z*_*t*_. Last, we note that Equation 11 is more likely to hold when the mixed layer is much deeper than the wave‐affected layer, $H\gg |Z_{t}|$, such that the bounds of integration remain sensible and residual term, *R*, remains relatively small.

These assumptions neglect many important processes in the upper ocean particularly during strong stabilizing conditions where the assumed stress and buoyancy decay functions are likely to be more nuanced. However, we feel that this attempt allows for a reasonably successful first order assessment of turbulent energetics in the mixed layer from measurements. Here, each term on the LHS of Equation 11 will be estimated with data collected from 10 months of mooring data, with the Stokes Production term following the same process as outlined for Eulerian shear in Section 2.2.

## 3 Data

Data used in this study were collected from the central mooring of the NASA Salinity Processes in the Upper‐ocean Regional Study (SPURS) field campaign (Farrar et al., 2015). The mooring was located at 25°N, 36°W, in the Atlantic, and recorded data from October 2012 to September 2013. The mooring consisted of a surface buoy with a suite of sensors to measure air/sea fluxes of heat, momentum, and freshwater (Fairall et al., 2003), as well as surface waves. Below the buoy was a heavily instrumented mooring line measuring temperature and salinity with vertical spacing near the surface of 3 m, and progressively coarser vertical resolution down to 120 m. Currents were measured on the mooring from 3 to 300 m with current meters and ADCPs.

The key element that enables this analysis is the measurements of TKE dissipation rate throughout the mixed layer, made possible by recent methods development with pulse‐coherent acoustic velocimeters (S. F. Zippel et al., 2021). For this study, pulse‐coherent ADCPs were used to estimate TKE dissipation rates at depths of 12.5, 21.5, 41.5, 61.7, 82, 101.6, and 121.6 m, with each burst sampled hourly for nearly the full mooring deployment.

**Figure 2:**
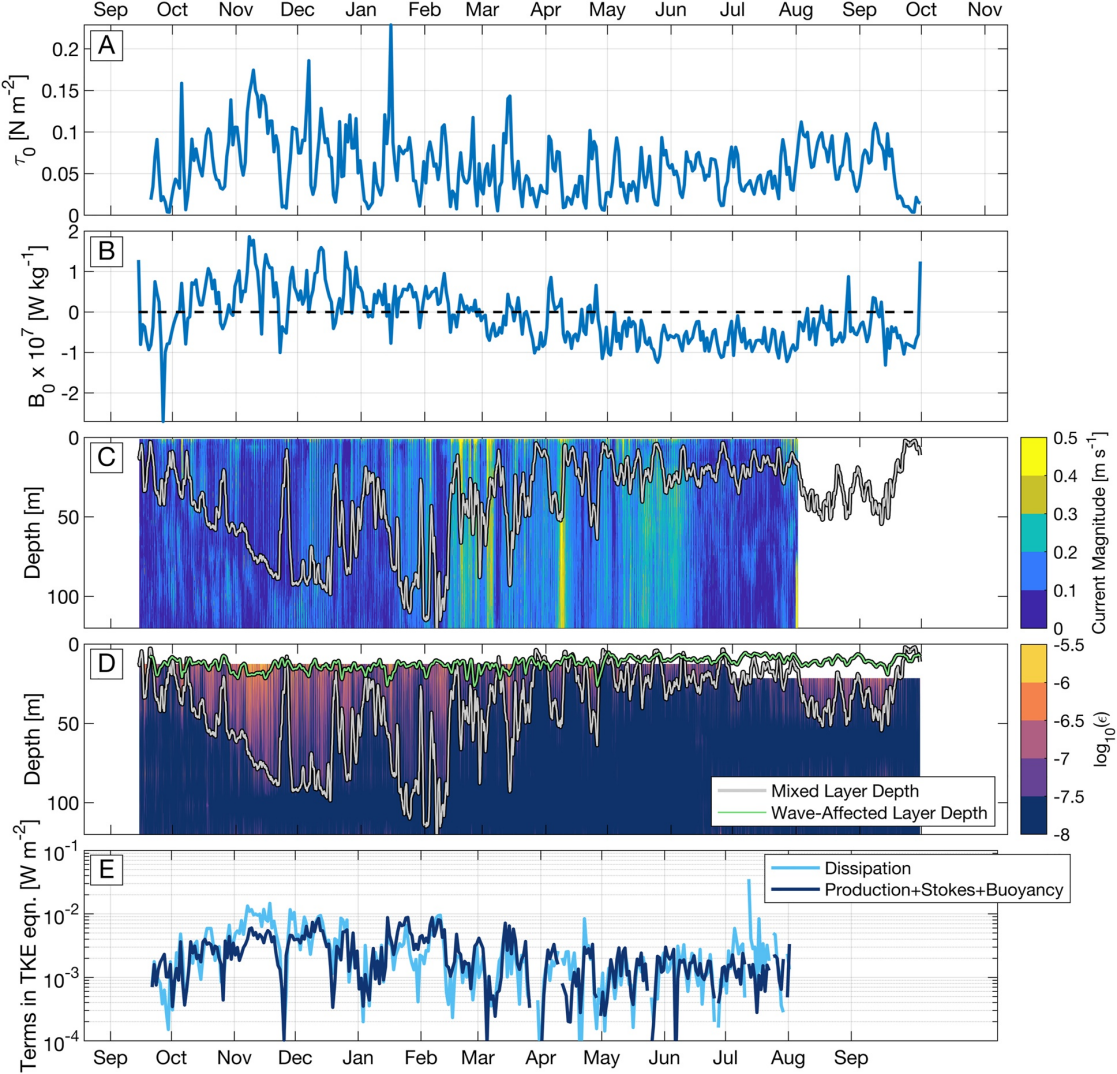
Time series of buoy‐measured products and estimates related to the terms in Equation 11 are shown above. Estimates of the ocean surface stress and the buoyancy flux (Equation 5, panel B) are shown as 24 hr averages. The magnitude of currents is shown in panel C with a 24‐hr mean mixed layer overlaid in gray. TKE dissipation rates are shown in panel D with both mixed layer depth and the estimated depth of the wave‐affected layer, *z*_*t*_ = −10*H*_*s*,*ww*_. Terms in the vertically integrated TKE equation estimated from measured surface fluxes, currents, and TKE dissipation rates are shown in panel (e) Terms in Panel E have been averaged in log space, due to the log‐normal distribution exhibited by TKE dissipation rate estimates.

Environmental conditions varied widely over the duration of the mooring deployment. During Fall 2012, 3‐week smoothed wind speeds and surface heat fluxes reached 8 m s^−1^ and −100 W m^2^, corresponding with significant deepening of the mixed layer to between 100 and 120 m (Farrar et al., 2015). Mean winds decreased in the early spring to less than 6 m s^−1^ and averaged surface heat fluxes switched from destabilizing to stabilizing. These changes corresponded to a decrease in mixed layer depths, with a strong diurnal cycle visible in the spring with mixed layer depths varying daily between nearly 1m to up to 50 m at night. The ADCP used to measure mean currents stopped recording in August 2013. The uppermost turbulence sensor stopped recording in early summer, while data from the lower sensors persisted until October of 2013. A time series of surface fluxes, currents, and TKE dissipation rates are shown in Figures 2a–2d.

### 3.1 Analysis

Terms in the TKE equation (Equation 11) are estimated based on bulk surface fluxes, and measurements of currents, waves, and TKE dissipation rates. The surface mixed layer depth was estimated as the depth where temperature difference with the surface is Δ*T* = 0.05°C. This threshold is intended to capture the depth over which density is nearly uniform, rather than the depth of active mixing (Brainerd & Gregg, 1995). Using a mixed‐layer depth defined with temperature only may result in some errors associated with the existence of barrier layers; however we do not expect these errors to significantly modify the results.

The surface production terms in Equation 11 are estimated using the buoy‐derived surface fluxes, the mixed‐layer depth, the vertical profile of currents, and the measured surface wave spectra. The wave stress was estimated using the (Plant, 1982) growth rate, *β*_*w*_, and the measured sea surface elevation spectrum. The stokes drift was estimated using Clarke & Van Gorder, 2018, appendix A on a 1 m‐spaced vertical grid, and the Stokes production was estimated following the procedure for the surface production with Stokes drift profiles used in the place of mean currents. Surface buoyancy fluxes are estimated using the assumed linear profile and the assumed double‐exponential decay profile of shortwave radiation.

TKE dissipation rates at depths of 12.5, 21.5, 41.5, 61.7, 82, 101.6, and 121.6 m were used to estimate the vertical integral of TKE dissipation (Equation 11). The log transform of TKE dissipation rates was linearly interpolated onto a 1m depth grid from *z* = *z*_*t*_ to 120 m using MATLAB’s *interp1* function. For the majority of the data set, the wave‐affected layer was deeper than the shallowest sensor. For cases where the *z*_*t*_ was shallower than the 12.5‐m instrument depth, the log transformed TKE dissipation rate profiles were extrapolated linearly to *z*_*t*_. This extrapolation represented a small fraction of the full depth profile with the exception of July 2013 when the 12.5 m instrument failed. Data from July 2013 and on were therefore excluded from further analysis. The TKE dissipation rate profile integration was performed on the interpolated grid using MATLAB's *trapz* function, from the mixed layer depth, *H*, to the bottom of the wave‐breaking layer, *z*_*t*_, for which we use *z*_*t*_ = −10*H*_*s*,*ww*_. Here, we differentiate the significant wave height, $H_{s}=4\sqrt{\int_{0}^{f_{N}}E\left(\omega \right)d\omega }$ from the significant height of the wind waves, $H_{s,ww}=4\sqrt{\int_{f_{e}}^{f_{N}}E\left(\omega \right)d\omega }$, where *f*_*e*_ is the energy weighted mean frequency of the sea surface elevation spectrum, and *f*_*N*_ is the highest frequency reported by the measurement package on the buoy. This height of the wind waves *H*_*s*,*ww*_ has been shown to better collapse a scaling for TKE dissipation rates in the wave‐affected layer (Gerbi et al., 2009), which is discussed further in Section 4.

Data where the wave‐affected layer was deeper than the mixed layer depth, |*z*_*t*_| > *H*, were excluded from analysis. These times caused non‐sensible buoyancy fluxes due to the shortwave radiation decay terms, which reverse sign when |*z*_*t*_| > *H*. These criteria also excluded data from strong stable buoyancy‐forced conditions, which were often associated with shallow stratification. These shallow, stable layers also break a number of assumptions summarized in Section 2.4 and are not the main focus of this work.

## 4 Results

A time series of the estimated terms of Equation 11 is shown in Figure 2e, where combined surface‐driven estimates of shear, stokes, and buoyancy forcing are compared with TKE dissipation rates. Values have been averaged in 24 hr blocks for visual clarity. For the majority of the year, the dissipation term is nearly in balance with the surface driven production and buoyancy terms, with values primarily ranging from 10^−4^ to 10^−2^ W m^−2^. The agreement is typically within the factor of 2 accuracy inherent to measurements of TKE dissipation rate (Moum et al., 1995; S. F. Zippel et al., 2021). The relatively good agreement here suggests terms left to the residual, *R* in Equation 11, including mixing and the wave‐affected layer residual, are indeed small compared with the estimated terms and/or compared to the errors associated with the dissipation measurement.

The worst agreement between the dissipation term and the surface forcing terms was in November, when the mixed layer deepened rapidly. Here, dissipation rates exceeded the estimated forcing by roughly a factor of 2. This is opposite to what would be expected if the mixing in the residual term, *R*, was large, because mixing would act as a further loss of kinetic energy, and the disagreement shows energy losses larger than the sources already. Worse agreement is also seen in late summer, when the 12.5 m ADCP used to estimate TKE dissipation rates failed. After early July, the poor agreement is attributed to an increase in number of extrapolated TKE dissipation rates used in the vertical integral.

**Figure 3:**
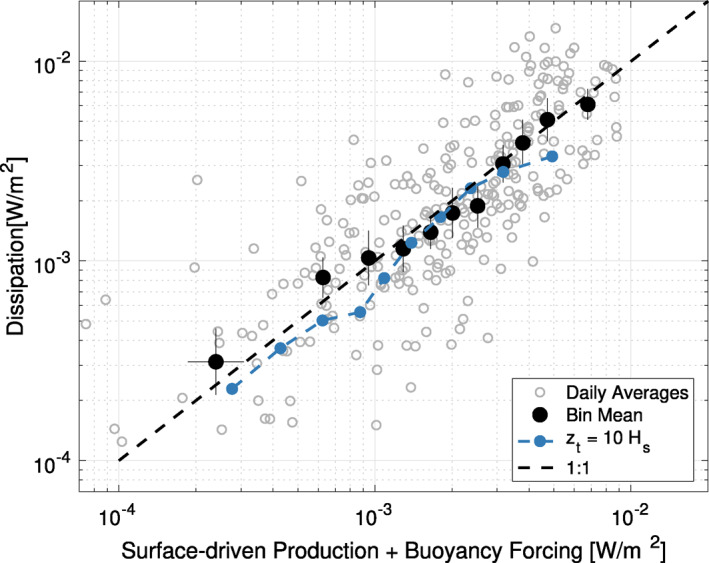
A direct comparison of the forcing and dissipation terms shown in Figure 2e is shown above for the full mooring duration. Gray open circles represent daily averaged estimates, with black circles showing binned means with vertical and horizontal bars showing log‐space 95% CI (twice the standard error). The dark blue line shows the effect of varying the upper bound of the TKE dissipation rate integral representing the transition depth of the wave‐breaking layer, *z*_*t*_ = −10*H*_*s*_. Solid black circles use *z*_*t*_ = −10*H*_*s*,*ww*_, which was suggested in Gerbi et al. (2009). The dashed black line shows 1:1 agreement between axes. Bins are chosen such that each contains an average of 25 daily averages; however this results in unequal bin spacing.

The agreement between forcing and integrated dissipation can be compared more directly in a scatterplot (Figure 3). Here, only data before the 12.5 m instrument failure are used. Good agreement is seen at all forcing levels (10^−4^–10^−2^ W m^−2^), with bin averaged TKE dissipation rates within 95% confidence intervals (twice the standard error) of the estimated forcing. Variability of the daily averaged values is high, but somewhat expected due to the large variance inherent to the TKE dissipation rate estimates (S. F. Zippel et al., 2021).

To assess the errors that might result from underestimating the depth of the wave‐breaking layer, we also show the effect of integrating to *z*_*t*_ = −10 *H*_*s*_ (Figure 3, dark blue). Here, using a deeper wave‐breaking layer still results in nearly balanced dissipation rates and total forcing, suggesting that Equation 11 remains valid as long as *z*_*t*_ is chosen to be sufficiently below the wave‐affected layer. This estimate is a conservative lower bound, since −10 *H*_*s*_ is likely deeper than the true wave‐affected layer depth. The available data was not sufficient to quantify the TKE balance within the wave‐affected layer, and future work is needed with full depth estimates of TKE dissipation rate to fully quantify surface‐layer turbulent energetics.

## 5 Discussion and Summary

Although the Stokes shear production was included in Equation 11, its estimated contribution to the TKE budget in the mixed layer (below the wave‐affected layer) was small, typically 1–2 orders of magnitude smaller than the mean shear production. The majority of Stokes drift shear is expected from very short waves which decay rapidly and therefore would be expected to be largest in the wave‐affected layer. Still, LES studies have suggested that the importance of Stokes effects lower in the mixed layer are subtle, enacted through enhanced downward transport and modified turbulence anisotropy (Li & Fox‐Kemper, 2020). In this context, the measurements here may still be in agreement with past LES results, as Stokes effects in the wave‐affected layer may play an important role in setting the vertical structure of currents throughout the mixed layer. Since the currents used in this analysis were measured directly (but not transport divergence, TKE dissipation rates, or stress in the wave‐affected layer), it is difficult to assess the impact of Stokes‐related terms.

### 5.1 Vertical Structure

Although not shown directly, the vertical structure of TKE dissipation rates was inconsistent with classic log‐layer shear scaling, $\epsilon ∼u_{*}^{3}/\kappa z$ during conditions with small buoyancy fluxes. This is somewhat surprising, given the general success of the vertically integrated Equation 11. The vertical structure of TKE dissipation rates was generally consistent with direct estimates of the local production term, made with the linearly decaying surface stress assumption. That is, for small buoyancy fluxes, $\epsilon \approx \left(1+z/H\right)\tau_{0}\cdot \left(d\bar{u}/dz\right)$. Therefore, it seems that the slab model (linearly decaying stress) is a reasonable first approximation, while the scaling for vertical shear $(d\bar{u}/dz)\approx u_{*}/\kappa z$ is not (here, *κ* is Von Karman’s constant). Although the estimated Stokes Production contribution was small here, it is possible that its effect on boundary layer turbulence is more important on setting the vertical shear, which has a secondary effect on the TKE budget. In this light, past work has shown changed turbulence anisotropy with decreasing Langmuir number, with an enhancement of the relative vertical turbulent motions (D’Asaro et al., 2014). Some recent work suggests that Langmuir circulations modify the pressure‐strain terms (Pearson et al., 2019), which act to redistribute energy between Reynolds stress tensor components and therefore might modify the local stress and shear without significantly increasing TKE.

Future work to better understand the nature of upper ocean turbulence would do well to focus on describing the vertical structure of shear, especially since numerous studies have failed to converge on the same scaling for TKE dissipation rates. Although the simplified linear decay of stress resulted in decent agreement in this study, future analysis would be greatly aided by direct estimates of ocean stress, in addition to a greater density of vertically distributed TKE dissipation rates (compared with the seven estimates over 120 m used in this study). Finally, direct measurements of the transport divergence terms, although challenging, would likely be a boon, as their importance is suggested by LES (Li & Fox‐Kemper, 2020; Pearson et al., 2019) and hinted at from limited field data sets (Scully et al., 2016).

To summarize,

1. We presented a conceptual framework to separate the turbulent energetics in the mixed layer from the near‐surface wave‐affected layer above
2. Using this framework, terms in the vertically integrated mixed‐layer TKE budget were estimated using 10 months of measured waves, currents, surface fluxes, and TKE dissipation rates all from the same mooring
3. Under this framework, vertically integrated TKE dissipation rates balanced surface‐driven production and buoyancy terms to within a factor of 2

## Data Availability

Open Research Data from the SPURS‐1 mooring, including TKE dissipation rates, are available through NASA's PO.DAAC https://podaac-tools.jpl.nasa.gov/drive/files/allData/insitu/L2/spurs1/mooring, and through WHOI's UOP website http://uop.whoi.edu/projects/SPURS/spurs1data.html. Code used for processing TKE dissipation rates in S. F. Zippel et al. (2021) is available at https://github.com/zippelsf/MooredTurbulenceMeasurements.
